# Correction: Long non-coding RNA LINC02163 accelerates malignant tumor behaviors in breast cancer by regulating the microRNA-511-3p/HMGA2 axis as a competing endogenous RNA

**DOI:** 10.32604/or.2024.051893

**Published:** 2024-08-23

**Authors:** CHENGLIN QIN, LINFANG JIN, JIA LI, WENZHANG ZHA, HUIMING DING, XIAORONG LIU, XUN ZHU

**Affiliations:** 1 Department of Thyroid and Breast Surgery, The Second Affiliated Hospital of Soochow University, Suzhou, Jiangsu 215000, China; 2 Department of General Surgery, The Fourth Affiliated Hospital of Nantong Medical College, Yancheng City No. 1 People’s Hospital, Yancheng, Jiangsu 224001, China; 3 Department of Pathology, Affiliated Hospital of Jiangnan University (Wuxi Fourth People’s Hospital), Wuxi, Jiangsu 214062, China; 4 Department of General Surgery, Affiliated Hospital of Nantong University, Nantong, Jiangsu 226001, China; 5 Department of General Surgery, The Second Affiliated Hospital of Jiaxing University, Jiaxing, Zhejiang 314000, China

In the article “Long non-coding RNA LINC02163 accelerates malignant tumor behaviors in breast cancer by regulating the microRNA-511- 3p/HMGA2 axis as a competing endogenous RNA” (Oncology Research, 2020, Vol. 28, No. 5, pp. 483–495. doi: 10.3727/096504020X15928179818438), there was an error in the processing of data. To further confirm our observation, we repeated multiple experiments involving in this study, including Flow Cytometry, Transwell Cell Migration and Invasion Assays, Xenograft Tumor Model, and Western Blotting. We have revised the figures to correct these errors. Corrected versions of the Figs. 2, 4, 5, 6, and 7 are provided. The corrections do not change any results or conclusion of the article. We apologize for any inconvenience caused.

**Figure 2:**
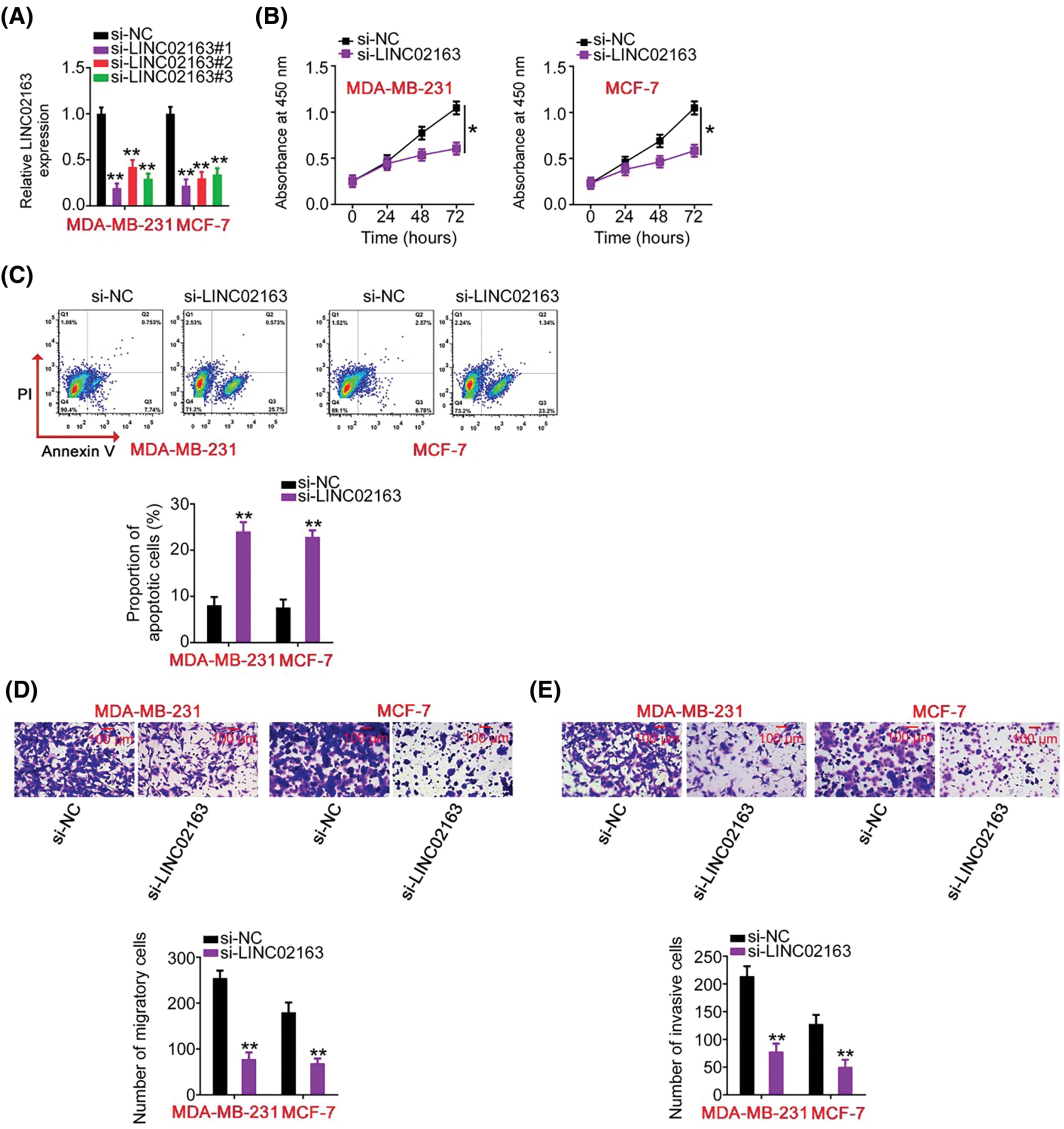
Impacts of LINC02163 silencing on the malignant properties of breast cancer cells *in vitro*. (A) Three siRNAs (si-LINC02163#1, si-LINC02163#2, and si-LINC02163#3) were designed to knockdown endogenous LINC02163 expression in MDA-MB-231 and MCF-7 cells. RT-qPCR verified the transfection efficiency. (B) Proliferation was analyzed using the CCK-8 assay in MDA-MB-231 and MCF-7 cells after LINC02163 silencing. (C) Flow cytometry was conducted to determine the apoptosis rate of LINC02163-deficient MDA-MB-231 and MCF-7 cells. (D, E) The migration and invasion capacities of MDA-MB-231 and MCF-7 cells after LINC02163 silencing were evaluated using Transwell cell migration and invasion assays. *p < 0.05 and **p < 0.01.

**Figure 4:**
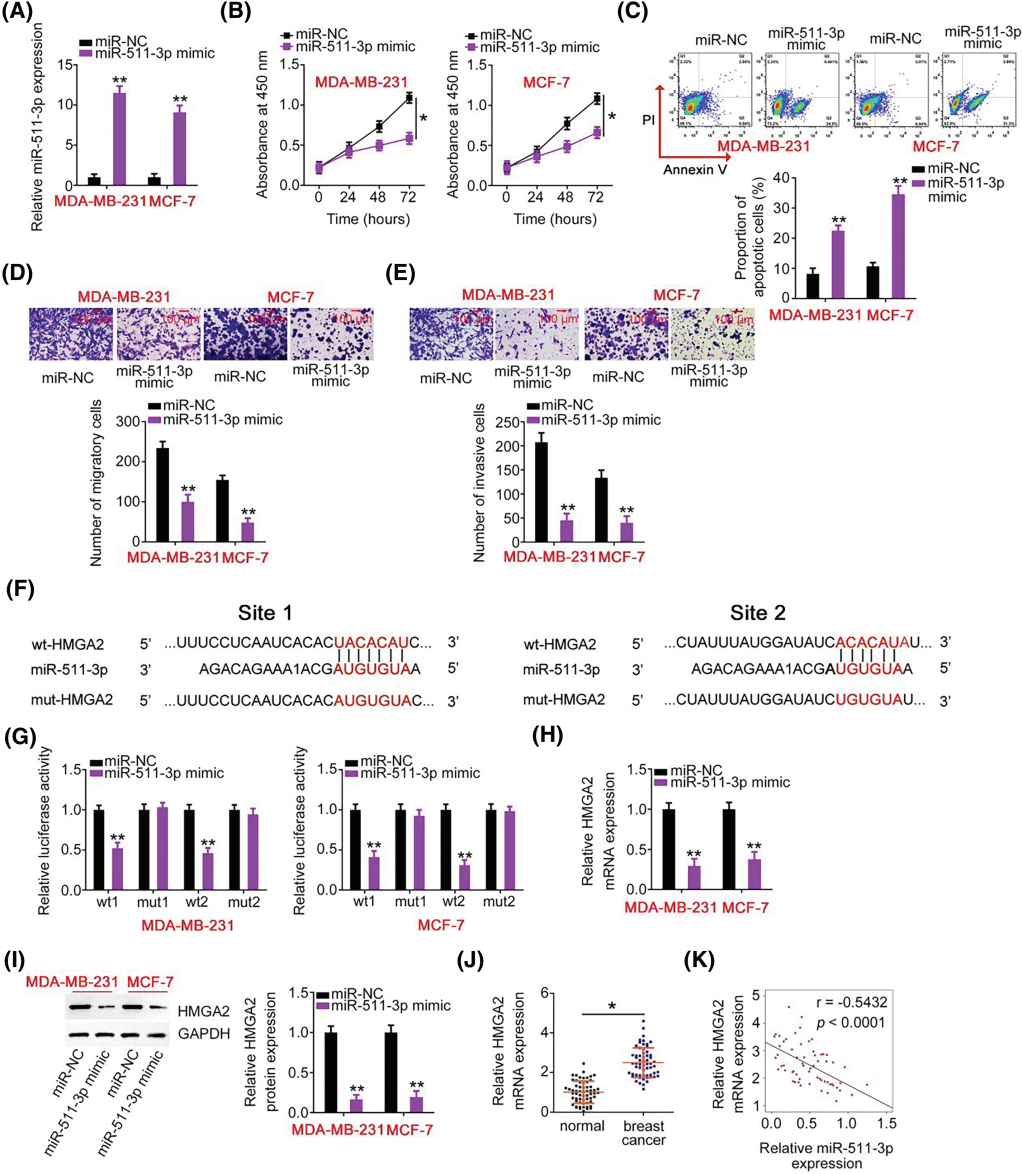
miR-511-3p acts as a tumor-suppressive miRNA in breast cancer and directly targets HMGA2. (A) The efficiency of miR-511-3p mimic transfection in MDA-MB-231 and MCF-7 cells was determined by RT-qPCR. (B, C) The effects of miR-511-3p upregulation on MDAMB-231 and MCF-7 cell proliferation and apoptosis were evaluated using CCK-8 assay and flow cytometry. (D, E) The migration and invasion capabilities of MDA-MB-231 and MCF-7 cells with miR-511-3p overexpression were detected using Transwell cell migration and invasion assays. (F) The wild-type binding site exists between miR-511-3p and HMGA2. Mutant binding sequences are also shown. (G) Luciferase activity was detected in MDA-MB-231 and MCF-7 cells after cotransfection with wt-HMGA2 or mut-HMGA2 reporter plasmids and miR-511-3p mimic or miR-NC. (H, I) HMGA2 mRNA and protein expression in miR-511-3p mimic-transfected or miR-NC-transfected MDA-MB-231 and MCF-7 cells were examined by RT-qPCR and western blotting, respectively. (J) HMGA2 mRNA expression was examined in 61 pairs of breast cancer tissues and matched adjacent normal tissues using RT-qPCR. (K) Spearman’s correlation analysis was applied to dissect the expression correlation between miR-511-3p and HMGA2 mRNA in the 61 breast cancer tissues (r = −0.5432, *p* < 0.0001). *p < 0.05 and **p < 0.01.

**Figure 5:**
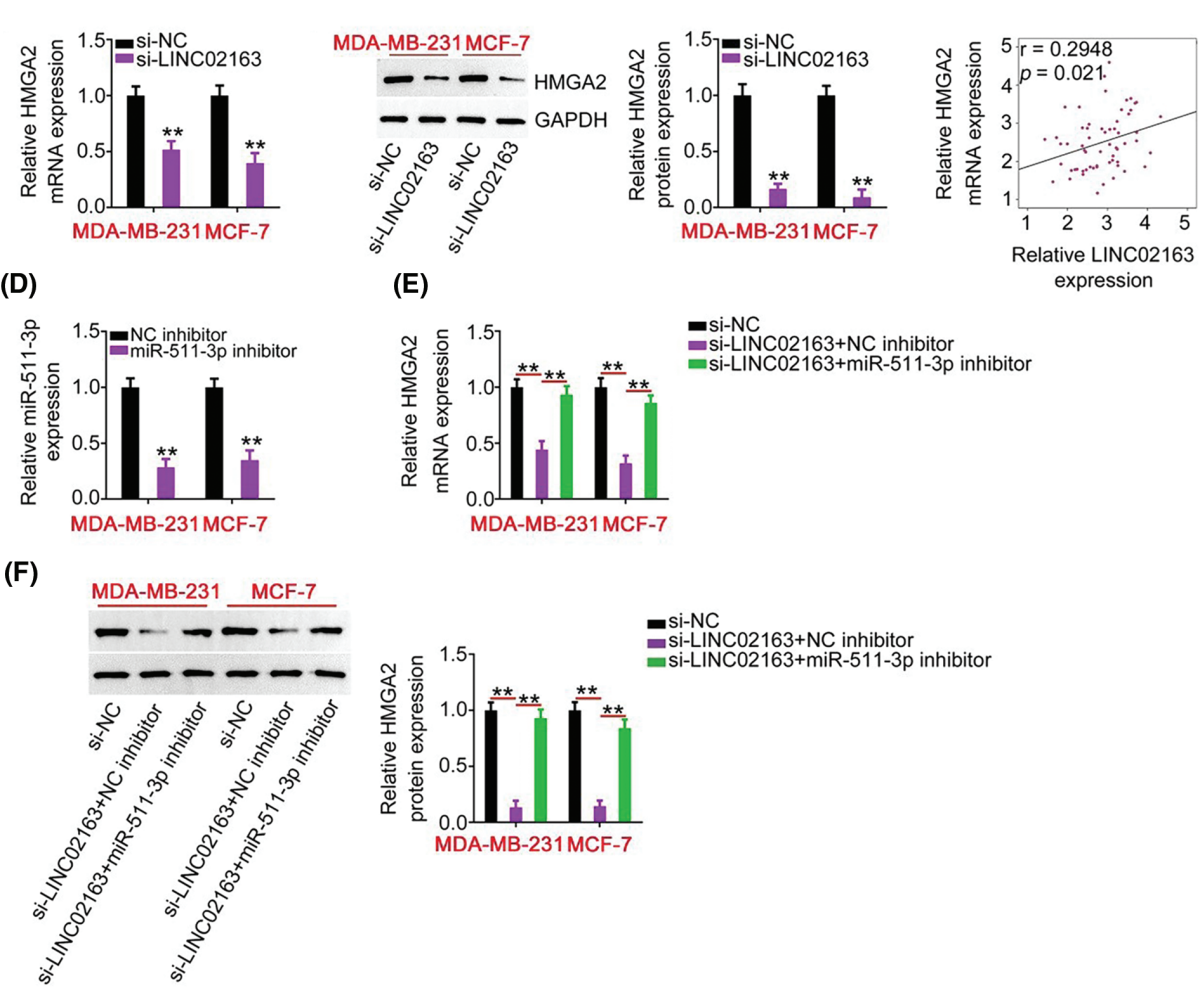
LINC02163 knockdown decreases HMGA2 expression in breast cancer cells through sponging miR-511- 3p. (A, B) The mRNA and protein levels of HMGA2 in LINC02163-depleted MDA-MB-231 and MCF-7 cells were analyzed using RT-qPCR and western blotting, respectively. (C) Spearman’s correlation analysis was performed to examine the correlation between LINC02163 and HMGA2 mRNA levels in 61 breast cancer tissues (r = 0.6914, *p* < 0.0001). (D) The knockdown efficiency of miR-511-3p inhibitor in MDA-MB-231 and MCF-7 cells was detected using RT-qPCR. (E, F) si-LINC02163 along with miR-511-3p inhibitor or NC inhibitor was cotransfected into MDA-MB- 231 and MCF-7 cells. The mRNA and protein levels of HMGA2 were detected using RT-qPCR and western blotting, respectively. **p < 0.01.

**Figure 6:**
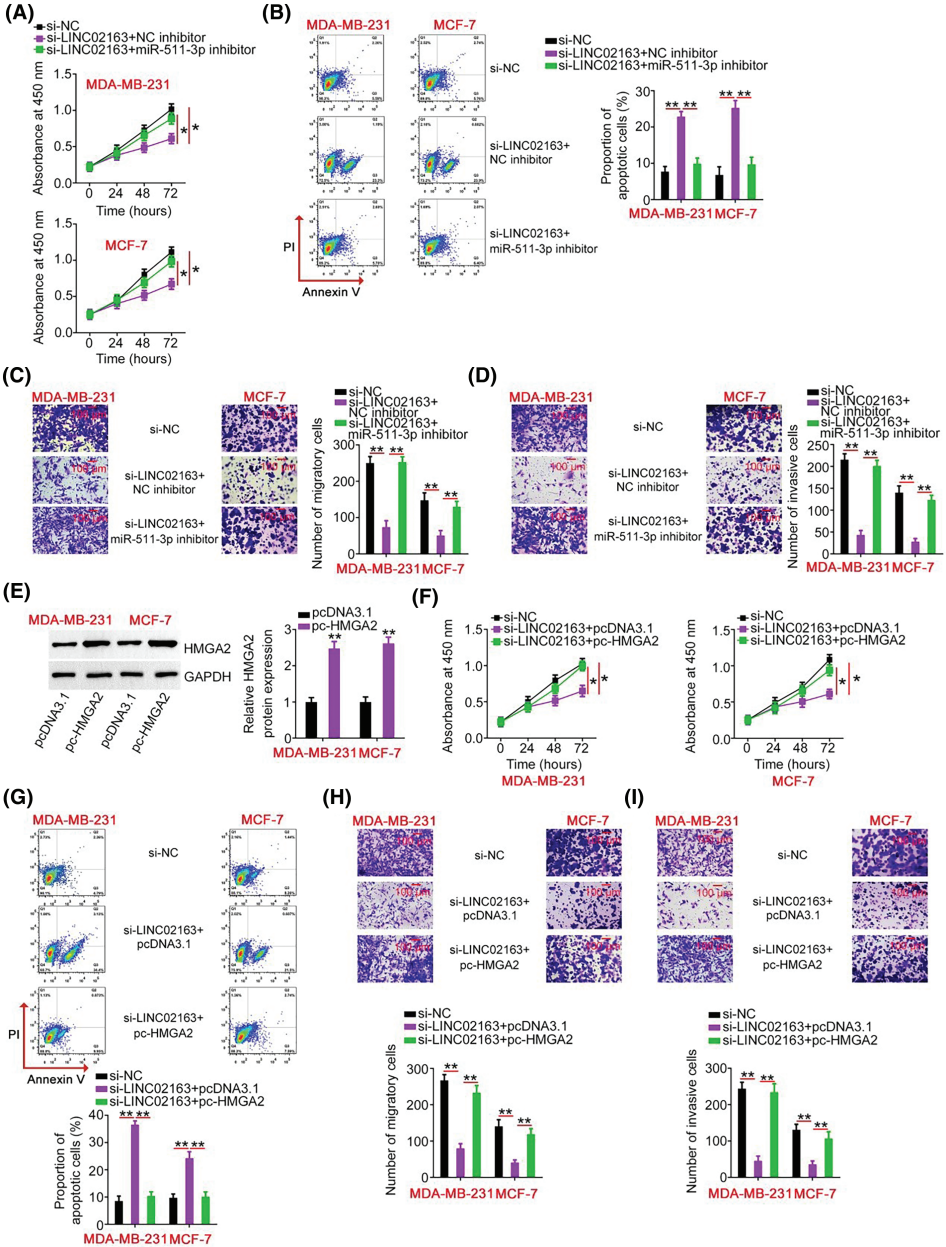
Elevation output of the miR-511-3p/HMGA2 axis abrogates the effects of LINC02163 knockdown in the malignant properties of breast cancer cells. (A, B) miR-511-3p inhibitor or NC inhibitor was introduced to MDA-MB-231 and MCF-7 cells in the presence of si-LINC02163. CCK-8 assay and flow cytometry were used to examine cell proliferation and apoptosis. (C, D) Migration and invasion of MDA-MB-231 and MCF-7 cells treated as described above were analyzed using Transwell cell migration and invasion assays. (E) The protein level of HMGA2 in MDA-MB-231 and MCF-7 cells following transfection of pc-HMGA2 or pcDNA3.1 was measured using western blotting. (F–I) si-LINC02163 in combination with pc-HMGA2 or pcDNA3.1 was transfected into MDA-MB-231 and MCF-7 cells. After transfection, cell proliferation, apoptosis, migration, and invasion were analyzed using CCK-8 assay, flow cytometry, and Transwell cell migration and invasion assays, respectively. *p < 0.05 and **p < 0.01.

**Figure 7:**
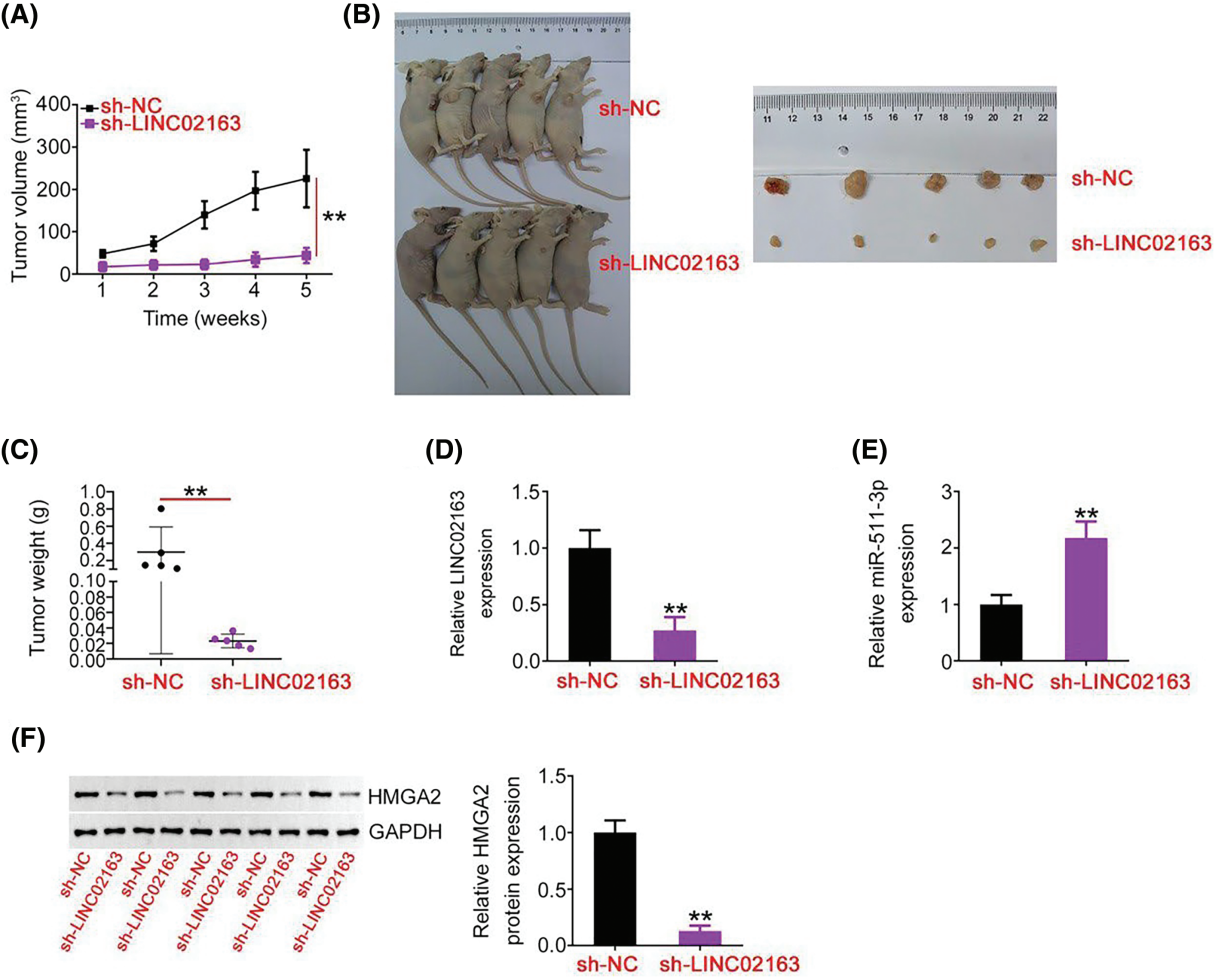
A reduction in LINC02163 expression attenuates breast tumor growth *in vivo*. (A) Growth curve of subcutaneous xenografts harvested from sh-LINC02163 and sh-NC groups. (B) Representative images of subcutaneous xenografts originating from MDA-MB-231 cells stably expressing sh-LINC02163 or sh-NC. (C) All mice were anesthetized 5 weeks after cell injection. Tumor xenografts were resected and weighed. (D, E) LINC02163 and miR-511-3p levels in the tumor xenografts removed from nude mice were detected using RT-qPCR analysis. (F) The protein expression of HMGA2 in the tumor xenografts was determined using western blotting. **p < 0.01.

The authors would like to correct the figures below:

**Table:** 

Page. No.	Exact Figure to be corrected	Correction
488	Fig. 2	Replace with new Fig. 2
490	Fig. 4	Replace with new Fig. 4
491	Fig. 5	Replace with new Fig. 5
492	Fig. 6	Replace with new Fig. 6
493	Fig. 7	Replace with new Fig. 7

The authors state that the scientific conclusions are unaffected. This correction was approved by the Oncology Research Editorial Office. The original publication has also been updated.

